# Weeneebayko Area Health Authority-ICES-Laurentian University Collaboration: Working together to support communities with Indigenous Health Research in the James and Hudson Bay Region, in Northeast Ontario, Canada.

**DOI:** 10.23889/ijpds.v7i3.2086

**Published:** 2022-08-25

**Authors:** Beth Rachlis, Kathleen Qu, Justice Seidel, Yantao Zhao, Kiana Seidel, Robert Gagnon, Sandra Kioke, Elaine Innes, Minnie Ho, Sujitha Ratnasingham, Jennifer D. Walker, Loretta Loon

**Affiliations:** 1 ICES; 2 Weeneebayko Area Health Authority (WAHA); 3 McMaster University; 4 Laurentian University

## Objectives

The James and Hudson Bay Region, consisting of six remote Indigenous communities, have experienced barriers accessing regional health data. To inform local health planning, the Minomathasowin Healthy Living department in the Weeneebayko Area Health Authority (WAHA), ICES and Laurentian University developed a Collaboration, to co-create enhanced Indigenous data stewardship.

## Approach

The Collaboration combines expertise in Indigenous knowledge with quantitative and qualitative analyses to develop relevant data intended for public dissemination. Through a community-driven and strength-based approach, local knowledge guides the direction of the research. Indigenous data governance principles are applied, supporting local data ownership, and supplementing local knowledge on population health issues. This ensures the development of research projects that have meaningful impacts. The Collaboration is part of a larger partnership and is continually engaging local Indigenous stakeholders. Protocols ensure research is done in a manner that respects and reflects community well-being and is undertaken in a good way.

## Results

The Collaboration is an ongoing, living initiative and has enabled WAHA to become a local hub for Indigenous stakeholders to obtain health data for their respective communities. It adheres to the importance of following protocols within Indigenous communities, acknowledging qualitative research activities can be undertaken at the community-level. Projects from this Collaboration identify and prioritize the most pressing health issues impacting the Region including mental health and addictions, COVID-19 surveillance, hospitalization trends, and the prevalence of lupus. The success of the Collaboration is demonstrated through increased requests from the Region to WAHA for support on health planning and decision-making. Data access barriers in the Region are being addressed through the combined expertise of the Collaboration and local knowledge. This approach is enhancing Indigenous data stewardship.

## Conclusions

The Collaboration advocates for Indigenous-led and -driven research that recognizes the value of combining local knowledge with quantitative and qualitative data analyses to put communities first. The Collaboration supports equitable data access and the development of relevant research projects. This is leading to sustainable, impactful health planning for the Region.

